# Are the Physiological Measures SpO _2_ and Heart Rate Biological Markers for Listening Effort and Fatigue?

**DOI:** 10.1055/s-0046-1827787

**Published:** 2026-10-14

**Authors:** Hemanth Narayna Shetty, Amruthvarshini Bhaskar, Teja Govindaraju

**Affiliations:** 1 Department of AudiologyJagadguru Sri Shivarathreeshwara (JSS) Institute of Speech and HearingMysuruKarnatakaIndia

**Keywords:** hearing aids, cognition, speech perception

## Abstract

**Purpose:**

To investigate whether physiological measures such as oxygen saturation (SpO _2_ ) and heart rate can reliably indicate listening effort and fatigue as well as possibly serve as objective alternatives to traditional subjective reports in the evaluation of the effects of digital noise reduction (DNR) in hearing aids.

**Methods:**

Through a repeated-measures design, we evaluated listening effort and fatigue in 35 participants with mild-to-moderately-severe sensorineural hearing loss using hearing aids, under DNR-on and DNR-off conditions. The participants performed a dual task for us to assess listening effort and fatigue, and physiological data (SpO _2_ and heart rate) were recorded with a pulse oximeter during the pretask, task, and posttask phases. Subjective ratings of listening effort, fatigue, attention, and concentration were obtained via self-report scales to assess DNR effectiveness and physiological marker sensitivity.

**Results:**

In DNR on, a significantly-higher score on repetition and recall was observed, along with reduced listening effort and reaction time, compared with DNR off. The posttest scores showed that the participants reported significantly less listening effort and fatigue, with improved attention and concentration in DNR on than in the pretest, whereas the opposite was true in the off condition. A notable difference in heart rates was observed between the pre- and post-test phases in DNR on. The median SpO _2_ levels significantly increased from the pre- to the post-task phases, with a notable difference during the task phase as well. Additionally, a negative correlation was found between fatigue and SpO _2_ in DNR on, while a positive relationship was noted between attention and SpO _2_ in DNR off.

**Conclusion:**

Physiological measures, particularly SpO _2_ , show preliminary promise as potential objective markers of listening effort and fatigue, aligning with behavioral and subjective outcomes, but this requires further validation.

## Introduction

Listening effort and listening-related fatigue have emerged as critical dimensions of auditory functioning, particularly in individuals with hearing impairment. Listening effort is defined as the deliberate allocation of mental resources to overcome obstacles in goal pursuit during auditory tasks. 1 In contrast, listening-related fatigue refers to the mental and physical exhaustion resulting from sustained or effortful listening. 2 These phenomena are especially pronounced in adverse listening conditions, such as background noise, 3 in which older adults with hearing loss exert greater cognitive effort to achieve speech understanding compared with those with normal hearing. 4 Listening in such adverse conditions results in fatigue, which can lead to a communication breakdown. 5 6 It can be explained by the Attention Capacity Model 7 , which demonstrates that the maximum cognitive resources are utilized to segregate noise from speech due to hearing loss, leaving fewer resources available for attending to, rehearsing, and retrieving the speech information. 8 9 10 Hearing aids improve audibility for individuals with hearing impairments. Digital noise reduction (DNR) in the hearing aid is designed to improve listening comfort by reducing background noise, and users often report increased comfort with it. 11 Studies 12 show that DNR does not always enhance speech recognition; it significantly reduces the listening effort, particularly under challenging listening conditions, and DNR has been shown to decrease attentional demands, improve focus, and reduce mental fatigue by lowering the cognitive load from background noise. 13 14 Behavioral evidence 12 of its impact on listening effort and fatigue remains mixed.

The growing interest in assessing listening effort and fatigue has led to the development of various measurement methods, including behavioral, subjective, and physiological approaches. The dual-task paradigm is one of the most widely-used behavioral methods. 1 It evaluates listening effort by having the participant perform a secondary task (such as reaction time [RT], recall) while completing a primary speech-perception task. Increased cognitive load in listening typically results in poorer performance on the secondary task, thereby reflecting the effort required. 15 The method has demonstrated good validity and sensitivity; 12 however, its implementation in clinical settings remains limited. It requires computer-based stimulus-delivery systems, standardized stimuli in the listener's language, and trained professionals to design and administer the tasks. 16 Thus, although promising, its practicality outside of research environments is constrained.

Physiological measures, particularly pupillometry, have also garnered attention for their ability to provide continuous and behavioral assessments of cognitive load. Studies 17 18 demonstrate that pupil dilation, a marker of cognitive strain, decreases when DNR is activated, indicating reduced effort. The pupil dilates in response to increased effort during listening tasks, offering a real-time window into the response of the autonomic nervous system (ANS) response. 19 However, the usefulness of pupillometry in clinical audiology is limited by its dependence on high-cost eye-tracking systems and controlled lighting conditions, which are typically available only in research laboratories and tertiary hospitals. In contrast, subjective questionnaires offer a feasible and cost-effective alternative to assess listening effort and fatigue. Instruments such as the National Aeronautics and Space Administration (NASA) Task Load Index (NASA-TLX), 20 the Listening Effort Questionnaire, 21 and the Speech, Spatial, and Qualities of Hearing Scale 22 have been widely used in audiological research. These tools are sensitive to the patient's own experiences and provide valuable insights into perceived effort. However, the inherent subjectivity of these measures and their susceptibility to response biases underline the importance of pairing them with behavioral assessments to enhance interpretability and reduce bias. 23

More recently, physiological markers such as the heart rate have been explored 24 as accessible, non-invasive indices of cognitive stress and fatigue during listening tasks. Listening effort has been associated with increased activity of the sympathetic nervous system, leading to variations in heart rate and skin conductance, and the results have shown an equivocal response. 25 Thus, another biofeedback measure—oxygen saturation (SpO _2_ )—, in conjunction with heart rate as a cross-check principle, is used to assess listening effort and fatigue. Continuous monitoring of heart rate and SpO _2_ is widely available in most hospitals and clinics, and it requires minimal technical expertise. Additionally, this approach may inform more practical and scalable methods for the clinical evaluation of listening-related cognitive demands in individuals with hearing impairment. Thus, a research question is formulated: Can the most feasible physiological measures in a hospital setting replace the subjective method, which is susceptible to response bias, with a valid and reliable behavioral measure of listening effort and fatigue? In this context, the present study aims to correlate physiological measures with subjective and behavioral measures under the DNR-on and DNR-off conditions.

## Methods

Correlative and comparative research designs were used to investigate whether the most feasible physiological measures could replace the subjective, reliable behavioral measures of listening effort and fatigue.

### Participants

The study included 35 native Kannada-speaking hearing aid users, aged 55–75 years (M = 67.02, SD = 4.98), with mild-to-moderately severe sloping sensorineural hearing loss. All participants had at least one year of hearing aid experience (M = 2.5 years, SD = 3.0) and were recruited from the clinical population of the Department of Audiology, Jagadguru Sri Shivarathreeshwara Institute of Speech and Hearing, Mysuru, Karnataka, India. Most participants reported no notable health problems, except for hearing loss. Only those using digital behind-the-ear (BTE) hearing aids bilaterally were considered eligible. The participants were required to be fluent in Kannada and have completed at least primary education. All individuals exhibited normal middle-ear function, as confirmed by A-type tympanograms. The participants also filled out the Montreal Cognitive Assessment (MOCA) to rule out significant cognitive dysfunction. The mean score of the participants was of 25.85 ± 1.51, (range: 25–30). Participants with a conductive component, neurological or psychiatric conditions, cognitive impairments, or any comorbid medical conditions were excluded from the study to ensure a homogeneous and clinically-stable sample. Furthermore, the participants were screened for cardiopulmonary conditions, smoking status (all non-smokers), medication use (excluding beta blockers or vasodilators, for example), and vascular factors via a self-report health questionnaire and review of medical records. Those with any positive history were excluded to minimize the influences on SpO _2_ and heart rate. The study procedures were reviewed and approved by the Jagadguru Sri Shivarathreeshwara (JSS) Institutional Ethical Committee (JSSMC/IEC/27102023/40 NCT/2023–24). Listening effort and fatigue were measured using subjective, behavioral, and physiological measures.

### Hearing-aid Programming

The participants used their own BTE hearing aids from multiple manufacturers (such as Phonak Naida, Oticon, Widex, and Aditivo). The hearing aid underwent electroacoustic measurements using the Drum hearing instrument test box (Inventis S.r.l.), confirming that equivalent input noise and harmonic distortion were within the American National Standards Institute (ANSI) S3.22 standards. The receiver in the canal hearing aid was fitted with a custom earmold. Two programs were tested: Program 1 used an omnidirectional microphone with feedback cancellation, while program 2 used a directional microphone with noise reduction. The systems automatically switched based on background noise. The directional and DNR features were verified using the front-to-back ratio and real-ear-aided response measurements. The settings were adjusted to meet the National Acoustic Laboratories-Non-linear 2 (NAL-NL2) prescriptive target, achieving an insertion gain of 5 dB from 0.25 to 2 kHz and within 10 dB at 4 and 6 kHz at an input of 65 dB SPL. The Speech Intelligibility Index was calculated for all participants, ranging from 0.75 to 0.88 (mean: 0.86 ± 0.07) in the signal-to-noise ratio of 50% (SNR-50) and dual-task conditions.

### Behavioral Measures

#### Listening Effort and Fatigue

The listening effort of the participants was measured using a dual-task paradigm in the DNR-on and -off conditions with hearing aids. It assesses speech recognition and listening effort, as indicated by accuracy scores and RTs.

#### Auditory Dual Task

At the beginning of the session, the subject was thoroughly briefed about the test procedure. Participants were comfortably seated in a quiet room, and the auditory stimuli were presented using a carrier phrase followed by the target word (e.g., 'Repeat the word...') delivered through the front speaker at 65 dB SPL. In contrast, the background noise, set to the SNR-50, was presented simultaneously through the rear speaker. Both loudspeakers were positioned 1 m apart at 0° (front) and 180° (back) azimuths.

#### Stimuli

The stimulus comprised 250 standardized monosyllabic nouns in the Kannada language. 26 Twenty blocks were created, each consisting of 5 words, accounting for 100 words (20 blocks of 5 words each). The remaining words were randomized for use in the visual (single-task) condition. The 250 words were nouns, including names, places, animals, things, and abstract concepts. Speech-shaped noise derived 27 from the words was mixed with the target word to achieve the desired signal-to-noise ratio (SNR). The SNR level was determined based on each participant's individual SNR-50 threshold, at which 50% speech recognition was achieved. Furthermore, the visual distractor was the word ‘STOP’ (font size: 48) in white font color against a red background displayed as a button to be clicked as soon as it appeared anywhere on the test screen. The nouns were used as a part of the primary task, and the distractor was used for the secondary task.

#### Procedure

a) In the dual-task paradigm, the primary task was the word-recognition task, while two secondary tasks were involved: the visual-distractor stimulus and the memory-recall activity. The participants listened to a speech delivered through a loudspeaker located at 0° azimuth, placed 1 m away from the listener, and the background noise presented from another loudspeaker located at 180° azimuth (behind the listener at ear level). The background noise level was fixed (55 dBA), and speech levels were chosen individually so that the average performance in the aided advanced condition was ∼ 50% correct (SNR-50).
  Five target words were sequentially presented in each block with an interstimulus interval of 5,000 ms. The subject was instructed to repeat the word they had heard and was encouraged to judge the word category displayed on the flashcard. The categorization of words was also scored based on the number of correct responses in their chosen category. Following each block, the visual distractor appears on the screen for 2,000 ms (duration of the visual display), and the participant must click the STOP button as soon as possible. Concurrently, the subject was asked to recall the five words presented in the primary task. The interblock interval was set at 20 s, after which the next block was presented. If the word was incorrectly identified during the initial presentation, recall was only scored correct if that same word was later recalled. This method increased the total number of items available for scoring compared with recall scores on only those words that were correctly identified. Likewise, 20 blocks were presented sequentially and randomized across participants. This process continued uninterrupted for ∼ 30 to 40 minutes. Two sets of 20 blocks were used to assess the listening effort under DNR-on and -off conditions, which were counterbalanced across participants to control for order effects, with half the sample starting in each condition.
b) Visual single task: This visual RT measure mimics the dual-task paradigm, in which participants listened to speech and noise at the same levels and in the same room configuration as during the dual-task testing. However, the participants were instructed to ignore the speech and noise (that is, not to repeat or try to recall any words) and focus solely on responding as quickly as possible to the visual prompt ‘STOP’. The participants completed 20 blocks in this condition.

#### Scoring

The total word recognition score, the average recall score, and the median visual RT were analyzed. The correctly-repeated words from 20 blocks were added to get the total word recognition score. The word recall score was obtained by averaging the number of correctly-recalled words from each block by the total number of blocks. The median visual RT throughout 20 blocks was calculated and used as the single-task visual RT (in seconds). The same procedure was repeated in both aided DNR-on and DNR-off conditions. Furthermore, fatigue was calculated by the following equation: Fatigue Score = Dual task Mean RT- Single task visual Mean RT / Dual task Mean RT (where RT = reaction time).

### Subjective Measures

The questionnaires were administered in a quiet room with adequate lighting. The researcher posed questions, and the responses to each question were recorded on printed paper and later analyzed. The subjective items listed below were administered before and after the measurement of listening effort and fatigue. Pre- and posttest scores were used to indicate the effect on these measures.

Fatigue and attention were assessed using the following questions:

Q1: How well can you maintain your focus and attention right now?
Scoring: From ‘very well-maintained attention’ (0) to 1 ‘cannot sustain attention’ (10).
Q2: How mentally drained are you right now?
Scoring: From ‘not mentally drained’ (0) ‘completely drained’ (10).
(Zero denotes no difficulty/fatigue experienced, while 10 represents the maximum fatigue experienced).

#### Speech, Spatial and Qualities of Hearing Scale (SSQ)

The subjective questions for the present study were drawn from the Speech, Spatial and Qualities of Hearing Scale (SSQ). 22 The direction of the rating scale was reversed in comparison to that of the original SSQ, so 0 always indicated no or minimal difficulty, while 10 indicated maximum difficulty.

The items are as follows:

Q3: ‘Do you have to concentrate very much when listening?’
Scoring: From ‘need not concentrate’ (0) to ‘concentrate hard’ (10).
Q4: ‘Do you have to put in a lot of effort to hear what is being said?’
Scoring: From ‘no effort in listening’ (0) to ‘a lot of effort in listening” (10).

### Physiological Tests

Listening effort, particularly during prolonged or demanding situations, can cause fatigue in the ANS), thereby affecting several vital functions. It indicates that an increased focus on listening activates the ANS, which controls the heart rate, breathing, and digestion; this activation can lead to fatigue and other physical effects. 28 The present study focused on heart rate (pulse) and SpO _2_ as important physiological metrics. These measures assess whether the differences observed in vital signs before, during, and after the test are related to variations in task performance, listening effort, and fatigue. The heart rate is determined by monitoring the time interval between peaks in the photoplethysmogram (PPG) signal, which reflects the changes in pulsatile blood volume with each heartbeat. 29 The measurement of SpO _2_ is made by evaluating the absorption of red and infrared light, and the estimation of its levels is performed through the ratio of the alternating current (AC) component (pulsatile) to the direct current (DC) component (non-pulsatile) of the PPG signal at each respective wavelength. 30 31 The pulse oximeter was positioned on one index finger (on the hand opposite the writing hand). This device emits infrared light through a sensor attached to the finger and measures the amount of infrared light reflected from the skin's surface. An Onyx Vantage 9590 (Nonin Medical Inc) finger pulse oximeter was used, with a sampling rate of 1 Hz and accuracy of ± 2% for SpO _2_ (70–100% range). No signal averaging was applied, and motion artifacts were controlled by seating participants in a quiet room and instructing them to minimize hand movements.

### Statistical Analyses

The data on behavioral, subjective, and physiological effort in listening were entered into the IBM SPSS Statistics for Windows (IBM Corp.) software, version 26.0, for the statistical analysis. The data were assessed for normality using the Kolmogorov-Smirnov test and the Levene's homogeneity of variance test. The results revealed that the data were not normally distributed ( *p* < 0.05). The data were subjected to descriptive analyses to derive the median and interquartile range values for the aforementioned measures. The Wilcoxon signed-rank test was used to compare data of behavioral and subjective measures from the DNR-on and -off conditions and from the pre- and postmeasures. The physiological measures (SpO _2_ and heart rate) obtained at three time points were subjected to the Friedman test. If significant, the Wilcoxon signed-rank test was performed with Holm's adjusted *p* -values for multiple comparisons (α = 0.05/3 = 0.017). The effect size was calculated using the rank-biserial correlation, with the standardized-value divided by the square root of the sample size: r=Z/ where Z is the standardized test statistic, and N is the total sample size. In addition, the Spearman's rank correlation was used to assess the relationships involving the physiological measures and the data on listening effort and fatigue.

## Results

### Behavioral Measures

To determine the impact of DNR on listening effort using a dual–task paradigm, data on word recognition, recall, and reaction time were analysed using the Wilcoxon signed–rank test. Participants performed better in the DNR–on condition, with higher median scores for word recognition (Mdn = 51.00, range = 36.00–66.00) and recall (Mdn = 2.17, range = 1.45–2.90) compared with the DNR–off condition (Mdn = 40.00, range = 25.50–54.50; Mdn = 1.65, range = 0.93–2.38, respectively). These differences were statistically significant for both word recognition [Z = −5.16, p < 0.001, r = 0.87, CI (0.986–1.680)] and recall [Z = −4.89, p < 0.001, r = 0.83, CI (0.842–1.536)], indicating reduced listening effort with DNR. Reaction time was faster in the DNR–on condition (Mdn = 1.57 s, range = 0.80–2.35 s) than in the DNR–off condition (Mdn = 1.69 s, range = 0.86–2.52 s), with a statistically significant difference [Z = −3.297, p = 0.001, r = 0.56, CI (0.285–0.979)], suggesting reduced cognitive load. Overall, DNR facilitated more efficient auditory processing in challenging listening environments. Furthermore, participants exhibited lower fatigue scores in the DNR–on condition (Mdn = 0.13, range = −0.02–0.28) than in the DNR–off condition (Mdn = 0.17, range = 0.02–0.32), with a significant difference [Z = ?5.045, p < 0.001, r = 0.85, CI (0.907–1.601)], indicating reduced mental effort with DNR-on.

### Subjective Measures

To evaluate the impact of DNR on the participants' experiences, questionnaire responses regarding concentration, listening effort, attention, and fatigue were analyzed using the Wilcoxon signed-rank test. While the scores were slightly higher in the DNR-off condition than in the on condition, the differences were not statistically significant, indicating that DNR did not notably affect the participants’ perceived concentration, listening effort, attention, or fatigue in the pre- and posttests.

Each questionnaire assessing subjective experience was analyzed by comparing pre- and posttest scores using the Wilcoxon signed-rank test, separately for the on and off conditions. The posttest scores showed a significant decrease compared with the pretest in concentration (Z = −4.919; *p* < 0.001; r = 0.83; CI = 0.69–0.91), attention (Z = −4.878; *p* < 0.001; r = 0.82; CI = 0.68–0.91) and fatigue (Z = −5.066; *p* < 0.001; r = 086; CI = 0.73–0.93) in the DNR-on condition. These results suggest that enabling DNR in hearing aids improved the participants' subjective perceptions of listening ease and comfort, supporting the notion that DNR can enhance everyday auditory experiences. Conversely, with the DNR turned off, the posttest scores were significantly higher than the pretest in concentration (Z = −5.130; *p* < 0.001; r = 0.87; CI= 0.76–0.93), listening effort (Z = −4.659; *p* < 0.001; r = 0.79; CI = 0.64–0.89), attention (Z = −5.136; *p* < 0.001; r = 0.87; CI = 0.76–0.93), and fatigue (Z = −4.874; *p* < 0.001; r = 0.82. CI= 0.68–0.91) ( Table 1 ).

**Table 1:** Median and range values for subjective measures pre- and posttest in the DNR-on and -off conditions

Questions	DNR-on				DNR-off			
	Posttest		Pretest		Posttest		Pretest	
	Median	Range	Median	Range	Median	Range	Median	Range
**Concentration**	3.00	1.00–6.00	4.00	2.00–7.00	5.00	3.00–8.00	8.00	6.00–10.00
**Effort**	5.00	2.00–8.00	3.00	1.00–6.00	5.00	3.00–8.00	4.00	2.00–7.00
**Attention**	3.00	1.00–6.00	5.00	3.00–8.00	6.00	4.00–9.00	5.00	3.00–8.00
**Fatigue**	3.00	1.00–6.00	4.00	2.00–7.00	5.00	3.00–8.00	5.00	3.00–8.00

Abbreviation: DNR, digital noise reduction.

### Physiological Measures

To assess the impact of DNR on physiological responses, heart rate and SpO _2_ levels were measured pre-, during, and posttask under the DNR-on and DNR-off conditions ( Table 2 ). Heart rate showed no significant difference between the measures. However, during task performance, SpO _2_ levels were significantly lower in the DNR-on condition than in the DNR-off condition (Z = −2.686; *p* = 0.007; r = 0.45; CI = 0.14–0.67). These results suggest that, while the heart rate was stable, SpO _2_ levels decreased in the DNR-on condition, indicating a possible physiological response to cognitive load.

**Table 2:** Physiological response in before, during and after measurements in the DNR-on and -off conditions

Questions	DNR-on						DNR-off					
	Pretest		During the test		Posttest		Pretest		During the test		Posttest	
	Median	Range	Median	Range	Median	Range	Median	Range	Median	Range	Median	Range
**Heart rate**	87.00	72.00–103.00	88.00	71.00–105.00	89.00	73.00–105.00	88.00	71.00–105.00	90.00	73.00–108.00	92.00	78.00–106.00
** SpO _2_**	89.00	86.00–93.00	91.00	89.00–94.00	94.00	91.00–97.00	93.00	90.00–97.00	95.00	93.00–98.00	96.00	94.00–99.00

Abbreviations: DNR, digital noise reduction; SpO _2_ , oxygen saturation.

The Friedman test was used to analyze heart rate at three time points (pre-, during, and posttest) under the DNR-on and -off conditions. In the DNR-on condition, although the mean ranks indicated a slight increasing trend in heart rate across the three time points, no statistically significant differences were observed (χ ^2^ (2) = 4.133, *p* = 0.127). Conversely, in the DNR-off condition, heart rate increased significantly across the three measurement time points (χ ^2^ (2) = 10.662, *p* = 0.005), with mean ranks progressively increasing from the pretest to the during-test and posttest periods. The Wilcoxon tests confirmed significant differences between the pretest and during phases (Z = −2.497; *p* = 0.013; r = 0.42; CI (= 0.10–0.65), as well as between the pre- and posttest (Z = −2.625; *p* = 0.009; r = 0.44 CI = 0.13–0.67); however, no significant difference was found between the posttest and during phases. These results indicate that participants experienced a significant increase in heart rate in the DNR-off condition, likely reflecting higher cognitive load or listening effort.

Under the DNR-on condition, there was a significant difference in SpO _2_ levels pretask, during the task, and posttask), as shown by the Friedman test (χ ^2^ (2) = 12.843; *p* = 0.002). The Wilcoxon Signed-Rank tests showed a significant increase from pre- to posttask (Z = −2.48; *p* = 0.013; r = 0.42; CI = 0.10–0.65) and from during the task to posttask (Z = −2.682; *p* = 0.007; r = 0.45; CI = 0.14–0.67), but no difference between pretask and during the task (Z = −1.386; *p* = 0.166) regarding SpO _2_ levels. This indicates recovery and improved SpO _2_ after the task, with posttask SpO _2_ being highest. In the DNR-off condition, a significant difference in SpO _2_ levels was noted across time points, as indicated by the Friedman test (χ ^2^ (2) = 8.824; *p* = 0 0.012). The Wilcoxon paired comparisons revealed a significant improvement from pre- to posttask (Z = −2.41; *p* = 0.016; r = 0.41; CI = 0.09–0.64), while other comparisons showed no significant differences.

### Relationship Involving Physiological, Behavioral and Subjective Measures

The relationship involving physiological scores during the dual-task paradigm and behavioral results in the DNR-on and -off conditions was evaluated using the Spearman's correlation. The results showed no significant correlation regarding physiological measures (heart rate and SpO _2_ ) and any of the behavioral outcomes (repetition, recall, and fatigue) in both conditions ( *p* > 0.05). These results suggest that physiological responses were not meaningfully associated with task performance, regardless of whether the DNR was activated. The correlation regarding posttest physiological scores and posttest subjective responses was examined in both conditions. In the DNR-on condition, a significant weak negative correlation was found between posttest SpO _2_ and fatigue (r = −0.354; *p* = 0.037), indicating that lower SpO _2_ was linked to higher levels of fatigue. Heart rate did not correlate with any subjective measures. Conversely, the DNR-off condition revealed no significant correlations involving posttest physiological measures (SpO _2_ and heart rate) and subjective ratings of concentration, listening effort, attention, or fatigue (all *p* > 0.05). Overall, these outcomes imply that the subjective assessment of fatigue was more closely associated with physiological measures when the DNR was activated, whereas this association was absent when the DNR was deactivated. The correlation regarding physiological findings during the dual-task paradigm and changes in subjective measures (pre- and postmeasurements) in both conditions was evaluated. In the DNR-on condition, there were no significant correlations regarding physiological measures and changes in subjective scores ( *p* > 0.05), indicating no meaningful connection involving heart rate or SpO _2_ during testing and fluctuations in concentration, listening effort, attention, and fatigue. On the other hand, in the DNR-off condition, a significant, weakly-positive correlation was observed regarding SpO _2_ during testing and changes in attention scores (r = 0.334; *p* = 0.050), indicating that higher SpO _2_ during the task was associated with improved attention. However, all other correlations involving heart rate or SpO _2_ during testing were weak and not statistically significant ( *p* > 0.05). The correlation regarding physiological findings during testing and the derived recall scores, calculated by subtracting the recall scores of the last 5 blocks from the first 15 blocks (and similarly for repetition scores), was analyzed in both conditions. In the DNR-on condition, a positive moderate correlation was identified involving SpO _2_ during testing and derived repetition scores (r = 0.527; *p* = 0.001), which was statistically significant. This finding indicates that higher SpO _2_ during dual-task performance was associated with better repetition performance. However, no significant correlations were observed involving heart rate during testing and derived recall and repetition scores in either condition.

## Discussion

The present study aims to assess whether physiological measures, such as SpO _2_ and heart rate, serve as biological markers for listening effort and fatigue. The activation of DNR in the study improved speech recognition and recall, and it reduced fatigue. It suggests that the DNR lessens the cognitive load required to process speech in noise. This finding is consistent with earlier studies showing that activation of noise reduction strategies attenuates background noise and improves the signal-to-noise ratio, thereby allowing more efficient allocation of cognitive resources for speech repetition, rehearsal, and subsequent recall 32 33 . These results are consistent with the effortfulness hypothesis, which postulates that cognitive resources allocated to attending and retrieving from memory require less effort to decode speech in a noise condition. 34 The study participants reported that using DNR-on in hearing aids reduced the listening effort.

Furthermore, a significant increase in SpO _2_ levels was observed from the during to the posttask phases when the DNR was enabled, suggesting physiological recovery. This improvement in SpO _2_ may indicate a reduction in autonomic strain following the cessation of effortful listening under reduced-noise conditions. This finding is consistent with earlier research suggesting that SpO _2_ may be sensitive to changes in cognitive workload 35 , with SpO _2_ -based measures demonstrating greater sensitivity to workload-related changes than heart rate in some experimental conditions. While SpO _2_ appears sensitive to subtle respiratory adjustments related to cognitive effort, heart rate may reflect broader autonomic responses that are not as easily influenced by improved auditory clarity alone. Activation of the DNR reduced the auditory processing load during the task, enabling listeners to maintain relatively-stable oxygenation throughout and facilitating a more rapid return to baseline physiological states following the task. The DNR decreases energetic masking and cognitive load, facilitating more efficient listening, which may prevent prolonged sympathetic activation. Although the SpO _2_ declined during the task, the recovery observed in the posttask phase indicated that noise-reduction algorithms buffered against prolonged physiological stress responses. 12 It represents a rebound to homeostasis once the listening challenge subsides. Research 36 evidence suggests that reduced listening effort is associated with decreased sympathetic arousal and faster physiological recovery.

Additionally, we calculated the difference between the average performance of the first 15 blocks and that of the last 5 blocks in the repetition task. This change in performance, as measured by the recognition score, was positively correlated with SpO _2_ , suggesting that increased repetition is associated with higher SpO _2_ levels. It suggests that better oxygenation during sustained listening supports speech perception accuracy, highlighting a potential interplay between physiological readiness and the cognitive-linguistic benefits conferred by noise-reduction features in hearing aids. 37 38 Thus, the DNR system may not only support cognitive performance but also foster faster posttask recovery in SpO _2_ . Therefore, the observed SpO _2_ trend emphasizes the significance of noise-management strategies in hearing aids to reduce the physiological strain and improve the listening comfort. 2

Additionally, due to the prolonged concentration and working memory demands, listening in noisy environments often results in increased mental fatigue. 2 However, DNR activation helps maintain mental endurance over time. When the DNR is enabled, this perceptual relief appears to directly contribute to lower subjective fatigue ratings during the dual task, which requires simultaneous speech processing and memory engagement. These findings also support the Framework for Understanding Effortful Listening (FUEL). 1 It suggests that fatigue is reduced when listening demands are within the available cognitive resources. Activation of the DNR may provide external support by reducing the cognitive cost of listening and enhancing the effectiveness of auditory-cognitive interactions for speech perception in complex tasks. Participants reported that activating DNR reduced their perceived fatigue and enabled them to maintain better attention and concentration while listening to speech in noisy environments. Due to sustained cognitive demand and listening effort, elevated physiological arousal resulted in a significant increase in heart rate from the pre to the posttest phases in the DNR-off condition. The participants were exposed to unfiltered background noise in the absence of DNR, which required them to allocate more attentional and cognitive resources to process speech stimuli. Sustained demand leads to an increase in heart rate by the activation of the sympathetic nervous system. 24

Furthermore, higher SpO _2_ was positively correlated with greater fatigue, suggesting that higher SpO _2_ is associated with greater cognitive strain and subjective exhaustion. Additionally, attention was positively correlated with SOP _2_ . This suggests that, under more effortful listening conditions, physiological resilience (that is, adequate SpO _2_ ) plays a greater role in sustaining cognitive engagement. Activation of the DNR in challenging auditory environments may lead to effortless listening, reduced fatigue, and increased engagement in communication, as supported by biological markers.

## Conclusion

Activation of the DNR in hearing aids significantly reduces listening effort, mental fatigue, and physiological stress. This is reflected in improved dual-task performance, increased SpO _2_ , indicating better oxygenation and recovery, and a lower heart rate, suggesting reduced autonomic arousal. The participants also reported improved listening comfort with the DNR. These findings suggest that SpO _2_ shows promise as a marker of listening effort and fatigue, whereas heart rate reflects general sympathetic activation. Overall, the DNR may enhance speech comprehension and support physiological well-being in challenging environments, though the results are exploratory and warrant larger-scale confirmation.

## Limitation

Baseline SpO _2_ values were lower than expected (median: 89–93%), potentially influenced by regional factors such as altitude or unmeasured subclinical conditions. This may limit generalizability and underscores the need for more rigorous physiological screening in future studies.
